# New "gold standard" for assessing myocardial oedema in STEMI?

**DOI:** 10.1186/1532-429X-15-S1-O70

**Published:** 2013-01-30

**Authors:** Elisa McAlindon, Xiaoming Bi, Chris Lawton, Mark C Hamilton, Nathan Manghat, Peter Weale, Chiara Bucciarelli-Ducci

**Affiliations:** 1 NIHR Cardiovascular Biomedical Research Unit, Bristol Heart Institute, Bristol, UK; 2 Siemens Medical Solutions, London, UK; 3 Siemens Medical Solutions, Chicago, IL, USA

## Background

The current "gold standard" CMR sequence for assessing myocardial oedema following STEMI is controversial. T2 Short-Tau Inversion Recovery (T2-STIR) is in widespread clinical use but can lack robustness. Steady state free precession oedema imaging (SSFP/ACUT2E) has emerging data to support it as a more reproducible method for oedema assessment. A novel T2 mapping method is also available to determine myocardial oedema. The potential benefit of this method is that the numerical output of the method is largely independent of myocardial motion, instrumental errors (e.g., surface coil normalisation methods). More recently, imaging early after gadolinium contrast administration (EGE) has been suggested as an alternative to detect myocardial oedema following STEMI. The aim of this study was to assess which of the CMR sequences for detecting myocardial oedema following STEMI is most robust.

## Methods

40 patients day 2 following STEMI were prospectively enrolled into the study. All patients had 2 CMR scans on the same day at least 6 hours apart. 3 slices (basal, mid cavity and apical) were repeated using all 4 sequences, with identical FOV, slice thickness and parameters optimised. 1920 images were analysed offline by 2 observers blinded to other sequence analysis results. The images were analysed using semi-automated software, and the myocardial oedema was expressed as a mass (g). Inter-observer, intra-observer and inter-scan agreement was assessed using the Bland Altman method. Variability was calculated as 1-intraclass correlation coefficient (ICC). The difference between sequences was assessed using a 1 way ANOVA. Qualitative analysis of the infarct related artery (IRA) territory with oedema for each sequence was compared against the IRA at the time of angioplasty.

## Results

The size of myocardial oedema significantly differs among the 4 sequences (p=0.0008). Bland Altman plots for inter-observer, inter-scan and intra-observer agreements were acquired for the 4 sequences. T2-STIR appears the least reproducible of the 4 techniques, with T2 mapping and EGE with the best intra, inter- observer and inter-scan agreement.

Inter-observer (T2-STIR bias -0.9 +/- 9.6; ACUT2E bias -2.5+/-6.9; T2 map bias -3.8+/-4.7; EGE bias -5.3+/-5.9).

Inter-scan (T2-STIR bias 1.5+/-5.8; ACUT2E bias -0.8+/-4.9; T2 map bias 3.1+/-4.0; EGE bias 1.1+/-4.9).

Intra-observer (T2-STIR bias 1.4+/-5.8; ACUT2E bias 0.6+/-4.7; T2 map bias 2.2+/-3.1, EGE bias 1.7+/-2.9).

Variability was lowest for T2 mapping, followed by EGE (Figure 2).

**Figure 1:**
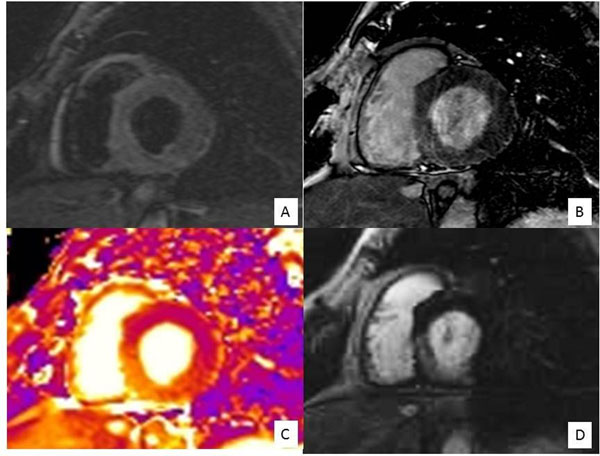
Short axis basal slice imaged with (A) T2 w STIR, (B) ACUT2E, (C) T2 mapping, (D) EGE. Myocardial oedema is best seen delineated on the T2 mapping and EGE.

**Figure 2:**
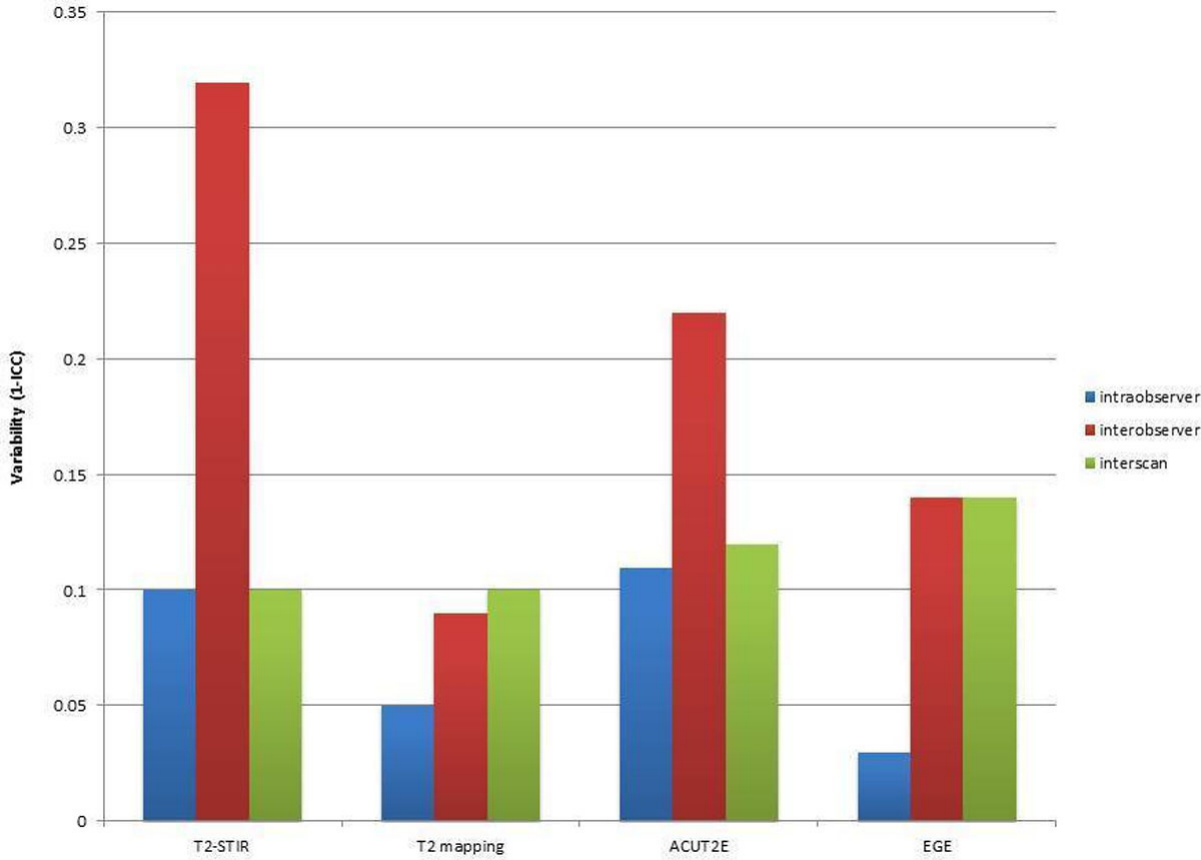
Intra-, inter-observer and interscan variability by oedema imaging sequence. Variability is calculated as 1-intraclass correlation coefficient (ICC).

On qualitative analysis, the correct IRA was identified in 83% cases by T2-STIR, 98% by T2 map, 88% by ACUT2E and 95% by EGE.

## Conclusions

The limitations of T2-STIR are well documented. This is the first study that systematically compared all available techniques to determine the most robust sequence for measuring myocardial oedema following STEMI. This study demonstrates that T2 mapping and EGE are robust methods to be used for surrogate endpoints in clinical studies.

## Funding

This work is funded by the NIHR Bristol Cardiovascular Biomedical Research Unit.

